# Neurodegenerative diseases and catechins: (−)-epigallocatechin-3-gallate is a modulator of chronic neuroinflammation and oxidative stress

**DOI:** 10.3389/fnut.2024.1425839

**Published:** 2024-08-01

**Authors:** Siying Li, Zaoyi Wang, Gang Liu, Meixia Chen

**Affiliations:** ^1^Hunan Provincial Engineering Research Center of Applied Microbial Resources Development for Livestock and Poultry, College of Bioscience and Biotechnology, Hunan Agricultural University, Changsha, China; ^2^Department of Neurology, The Yuhuan People’s Hospital, Taizhou, Zhejiang, China

**Keywords:** catechins, (−)-epigallocatechin-3-gallate, neurodegenerative diseases, Alzheimer’s disease, Parkinson’s disease

## Abstract

Catechins, a class of phytochemicals found in various fruits and tea leaves, have garnered attention for their diverse health-promoting properties, including their potential in combating neurodegenerative diseases. Among these catechins, (−)-epigallocatechin-3-gallate (EGCG), the most abundant polyphenol in green tea, has emerged as a promising therapeutic agent due to its potent antioxidant and anti-inflammatory effects. Chronic neuroinflammation and oxidative stress are key pathological mechanisms in neurodegenerative diseases such as Alzheimer’s disease (AD) and Parkinson’s disease (PD). EGCG has neuroprotective efficacy due to scavenging free radicals, reducing oxidative stress and attenuating neuroinflammatory processes. This review discusses the molecular mechanisms of EGCG’s anti-oxidative stress and chronic neuroinflammation, emphasizing its effects on autoimmune responses, neuroimmune system interactions, and focusing on the related effects on AD and PD. By elucidating EGCG’s mechanisms of action and its impact on neurodegenerative processes, this review underscores the potential of EGCG as a therapeutic intervention for AD, PD, and possibly other neurodegenerative diseases. Overall, EGCG emerges as a promising natural compound for combating chronic neuroinflammation and oxidative stress, offering novel avenues for neuroprotective strategies in the treatment of neurodegenerative disorders.

## 1 Introduction

**Figure 1:**
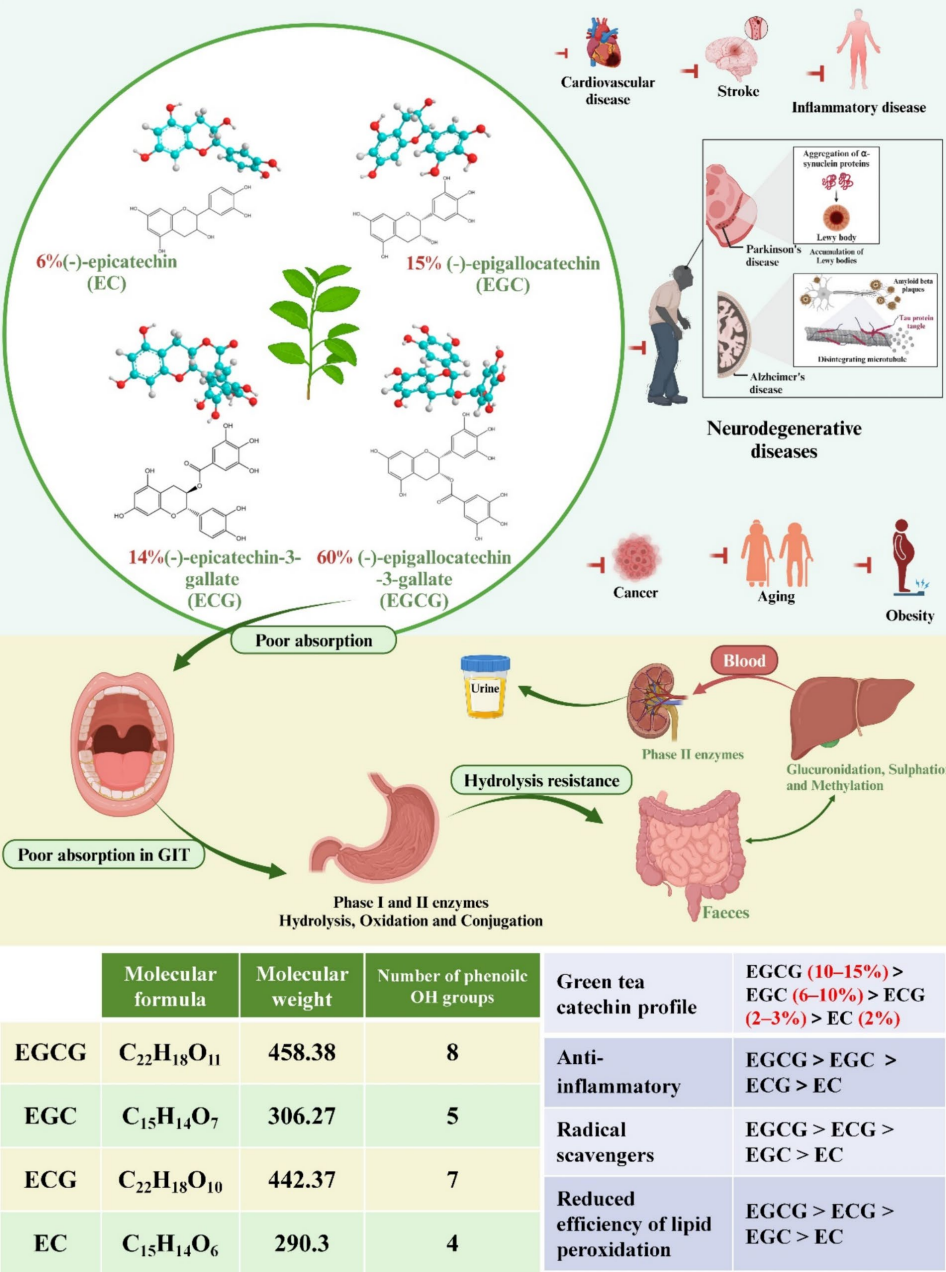
The chemical structures of four common green tea catechins are depicted. Their potential as therapeutic agents for common diseases is discussed. Additionally, the absorption and metabolism of green tea catechins are explored, accompanied by diagrams illustrating the absorption process across various organs of the body. Molecular formula, molecular weight, number of phenoile OH groups of four common catechins. Comparison of the four common catechins in green tea catechin profiles, anti-inflammatory, radical scavengers, and reduced efficiency of lipid peroxidation effects.

Catechins, a class of physiologically active phytochemicals, are commonly found in the fruits and leaves of various plants, including tea, apricots, cherries, peaches, blackberries, strawberries, blueberries, raspberries, and cocoa (1). Research indicates that catechins possess numerous health-promoting properties, notably benefiting cardiovascular disease, metabolic syndrome, diabetes, cancer, stroke, and neurodegenerative diseases (Figure 1) (2–9). As predominant polyphenols in tea, constituting approximately 30% of the dry mass of tea leaves, catechins serve as key functional components. Major green tea polyphenols encompass (−)-epicatechin (EC), (−)-epicatechin gallate (ECG), (−)-epigallocatechin (EGC), and (−)-epigallocatechin gallate (EGCG) (Figure 1) (10, 11). EGCG, the most abundant among green tea catechins at 60%, garners significant interest due to its broad spectrum of benefits elucidated in clinical trials, animal studies, and cell culture research (12). The molecular weight of EGCG is 442.37. Mechanisms underlying EGCG’s multifaceted health effects include antioxidant properties, anti-inflammatory activity, interactions with plasma membrane proteins, activation of second messenger and signaling pathways, modulation of metabolic enzymes, and promotion of autophagy (13–15).

Neurodegenerative diseases manifest through the gradual and progressive degeneration of nerve cells in defined regions of the brain and spinal cord, leading to functional impairment. Prominent examples encompass Alzheimer’s disease (AD), Parkinson’s disease (PD), Huntington’s disease (HD), and amyotrophic lateral sclerosis (ALS) (16–18). Although the specific cellular and molecular mechanisms vary across these diseases, common features include oxidative stress, mitochondrial dysfunction, DNA damage, protein aggregation, and neuroinflammation (18, 19). Notably, chronic neuroinflammation and oxidative damage represent shared pathological hallmarks among all neurodegenerative diseases (20, 21). Neuroinflammation serves as a common defense mechanism to protect the brain by removing or inhibiting various pathogens (22). This inflammatory response plays a crucial role in facilitating tissue repair and preserving tissue homeostasis (23). Typically, neuroinflammation abates upon successful tissue repair or pathogen clearance (22, 24). However, when the inflammatory stimulus persists, chronic neuroinflammation ensues (22, 25). Various factors contribute to sustained inflammatory responses, including protein aggregation, systemic infections, gut microbiota dysbiosis, aging, and genetic mutations. Prolonged activation of microglia and astrocytes, key players in neuroinflammation, can precipitate neurodegenerative diseases (26–28). Furthermore, neurons exhibit heightened susceptibility to oxidative damage, attributed to their elevated content of unsaturated fatty acids, rendering them susceptible to free radical attack and peroxidation. Additionally, increased levels of iron in specific brain regions further augment neuronal vulnerability to oxidative stress (29). Consequently, interventions targeting anti-neuroinflammatory and antioxidant pathways hold particular significance in the context of neurodegenerative diseases.

EGCG, a natural polyphenol abundant in green tea, exhibits promising neuroprotective properties attributed to its potent anti-inflammatory and antioxidant activities (12). Accumulating evidence underscores its therapeutic potential in the prevention and treatment of neuroinflammatory and neurodegenerative disorders (30). EGCG demonstrates notable neuroprotective efficacy by modulating signals implicated in autoimmune responses, enhancing interplay between the nervous and immune systems, and effectively attenuating inflammatory processes. Furthermore, EGCG exhibits iron chelation capabilities, scavenges free radicals, and exerts significant antioxidant effects, as evidenced by pertinent studies (31). Therefore, this review comprehensively explores the role of EGCG in various neurodegenerative conditions, particularly AD and PD, with a focus on elucidating its molecular mechanisms underlying anti-neuroinflammatory and antioxidant actions.

## 2 Antioxidant and anti-inflammatory effects of EGCG

Multiple investigations have substantiated the beneficial impact of green tea on neurodegenerative disorders. For instance, Shinichi Kuriyama et al. studied 1,003 elderly individuals aged over 70 years to assess the influence of green tea intake on cognitive function (32). Their findings revealed that subjects consuming more than 100 mL of green tea twice daily exhibited reduced susceptibility to neurodegenerative diseases (32). Similarly, Hu et al. conducted a 13-year longitudinal study involving nearly 30,000 Finnish adults, demonstrating that individuals consistently consuming over 600 mL of green tea daily exhibited a diminished risk of developing PD (33). These observations underscore the association between green tea consumption and a lowered incidence of neurodegenerative conditions.

**Figure 2:**
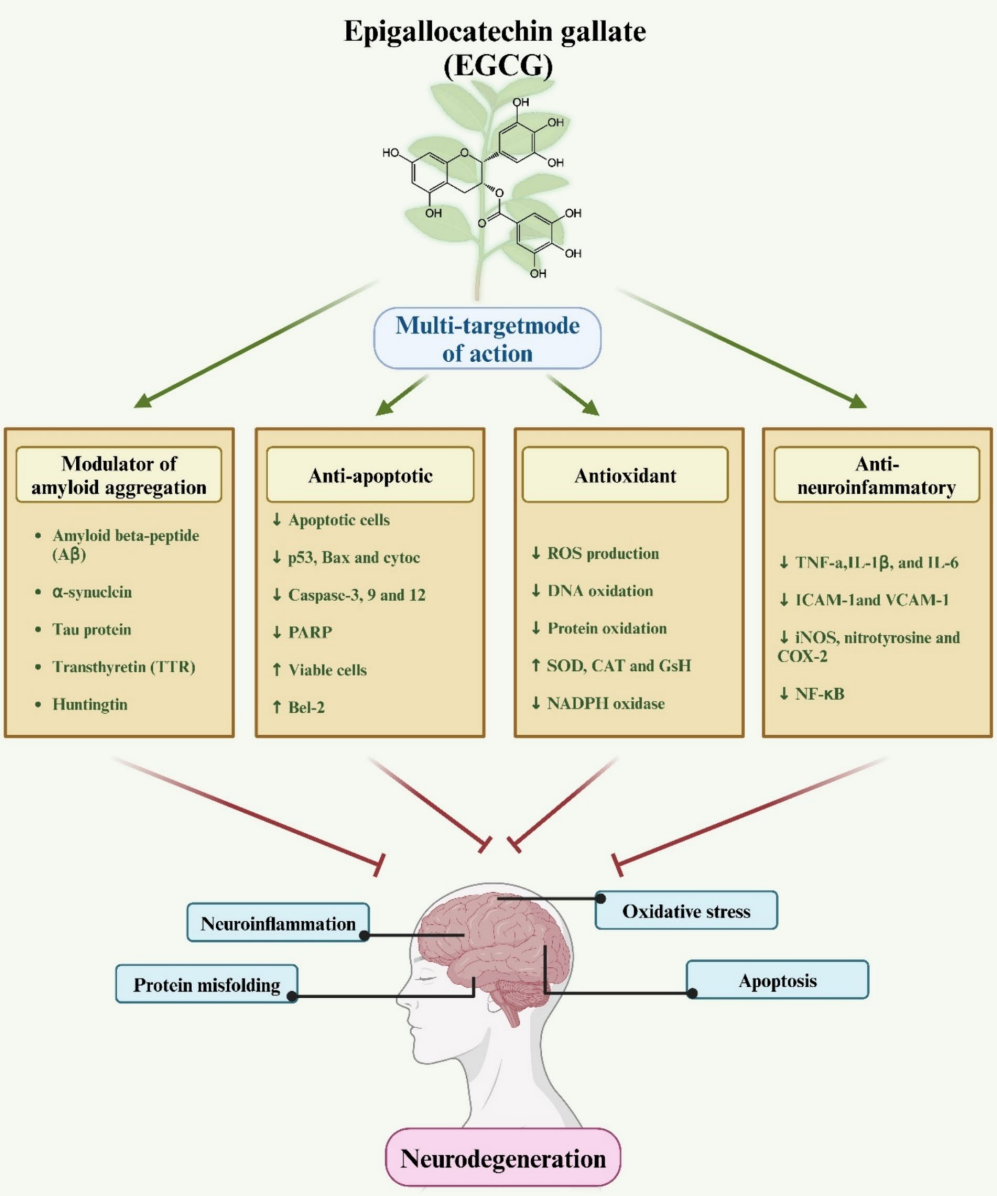
A schematic representation elucidates the role of EGCG in neuroprotection. The diagram illustrates how EGCG exerts antioxidant, anti-inflammatory, and anti-apoptotic effects via various molecular mechanisms, thereby conferring protection against neurodegenerative diseases.

The health-promoting bioactive components of green tea catechins include a wide range of isomers, the most representative of which are mainly four (EGCG, ECG, EGC and EC), with EGCG accounting for the vast majority of green tea research (34, 35). The biological action of the molecule will be determined by its chemical structure. EGCG (C_22_H_18_O_11_) is a catechin flavanol, specifically a gallate ester formed by the condensation of gallic acid with the (3R)-hydroxyl group of (−)-epigallocatechin, labeled A, B, C, and D (Figure 2) (36). The pentacosanoyl group esterification on Carbon −3 of the C-ring, along with hydroxyl groups on Carbon −3′, −4′, and − 5′ of the B-ring, underlie EGCG’s robust antioxidant activity compared to other catechins. The D- and B-rings contribute to its reactive oxygen species (ROS) neutralizing properties, with the D-ring further enhancing its anticancer and anti-inflammatory attributes. EGCG has seven hydroxyl groups in its aromatic ring. The location and number of hydroxyl groups on the ring determines its biological activity, giving EGCG greater antioxidant properties than EGC or EC, as well as water solubility, making EGCG highly permeable to the blood–brain barrier (BBB) (37). EGCG has been reported to cross the BBB within 0.5 h. Moreover, EGCG features two structures—the ortho-3′,4′-dihydroxy moiety and the 4-keto, 3-hydroxyl, or 4-keto, and 5-hydroxyl moiety—that can chelate metal ions, thereby neutralizing their activity. In essence, EGCG’s distinctive chemical structure and composition confer potent antioxidant and anti-inflammatory properties, suggesting potential benefits in select neurodegenerative disorders (38).

Following oral administration, EGCG undergoes limited absorption by the intestines, resulting in minimal entry into the bloodstream and tissues (39). The constrained bioavailability of orally administered EGCG arises from factors including extreme pH conditions, digestive enzymes, and EGCG’s restricted membrane permeability within the intestinal wall (9). Within the body, EGCG undergoes extensive biotransformation via sulfonation, glucuronidation, and methylation reactions (39). Its half-life is approximately 3.9 h, with complete metabolism occurring within 24 h (40). Furthermore, the biological effects of EGCG are contingent on concentration levels. Plasma concentrations ≤10 μM elicit antioxidant, anti-inflammatory, and insulin-sensitizing effects. Conversely, plasma EGCG levels exceeding 10 μM may induce pro-oxidant activity, augmenting autophagy and cell death, and are commonly employed in tumor therapy (41).

### 2.1 Anti-chronic neuroinflammatory effects of EGCG

Neuroinflammation serves as a protective mechanism within the nervous or central nervous system (CNS) against various threats including infections, toxic metabolites, autoimmunity, and traumatic brain injury, with the aim of eliminating harmful substances and damaged tissues (42). This process entails the activation of glial cells, which serve as neuroprotective agents by removing endogenous and exogenous substances while safeguarding themselves from ROS (43). Notably, microglia, as ubiquitous innate immune cells in the CNS, are pivotal contributors to neuroinflammation, participating in both anti-inflammatory and pro-inflammatory responses (44). The anti-neuroinflammatory properties of EGCG primarily involve the inhibition of microglial activation and the modulation of pro-inflammatory cytokine expression (45). The pro-inflammatory or neuroprotective functions of microglia are contingent upon their activation status (46). Pathogens or cellular debris induce heightened expression of pro-inflammatory cytokines such as IFNs and LPS, prompting microglial activation from a resting state (47). Activated microglia upregulate pro-inflammatory mediators including IL-1β, IL-23, TNF-α, IL-6, NO, and SOC3 via NF-κB and STAT1 pathways (48). In neuroinflammation, activated microglia sustain the release of pro-inflammatory cytokines, perpetuating chronic inflammation and generating cytotoxic molecules such as ROS and RNS (49). Extensive scientific evidence underscores the role of persistent inflammation in promoting neurodegenerative disorders. Conversely, neuroprotective microglia activated by IL-13, IL-10, and IL-4 secrete various factors associated with neuroprotection and tissue repair, including TGF-β, Chi3l3, Arginase 1, Ym1, IGF-1, and Fzd1 (48).

The effects of EGCG on microglia encompass: (1) Modulation of microglial activation under inflammatory conditions, primarily within the M1/M2 spectrum (50). M1 microglia release neurotoxic and inflammatory factors such as IL-6, IL-1β, and TNF-α, contributing to neuronal damage and death, while M2 microglia secrete neurotrophic factors including BDNF, IL-4, and IL-10, fostering neuronal growth and protection (51). EGCG downregulates M1 markers (IL-6, TNF-α, and IL-1β) and upregulates M2 markers (IL-10 and NQO1) in microglia, thereby modulating the M1/M2 ratio and mitigating neurotoxicity and neuronal damage arising from microglial hyperactivity (13). (2) EGCG induces M1 polarization via various signaling pathways including TLR4/NF-κB, JAK2/STAT3, TLR2, TLR4, JNK/P38, thereby suppressing the activation of inflammatory vesicles and reducing microglial inflammation and neurotoxicity (13). (3) Voltage-gated proton channels play a pivotal role in microglial NADPH oxidase-dependent ROS generation (52). EGCG impedes proton channel function in microglia without affecting channel gating processes. This inhibition of proton channels constitutes a significant mechanism through which EGCG suppresses microglial activation and neurotoxicity (53). (4) Neuronal injury or neuroinflammation triggers microglial activation, leading to NO production. NO reacts with cysteine thiols, resulting in protein S-nitrosylation, which regulates various cell signaling and protein activities, including protein misfolding and mitochondrial apoptosis. EGCG attenuates protein S-nitrosylation in activated microglia (54). In summary, EGCG mitigates excessive inflammatory responses and neurotoxicity induced by inflammation by inhibiting inducible NO synthase activity, reducing oxidative stress levels, and modulating the M1/M2 ratio in microglia.

### 2.2 Antioxidant effects of EGCG

EGCG, a significant natural antioxidant, demonstrates efficacy in neutralizing ROS like hydrogen peroxide, superoxide anions, and hydroxyl radicals (55). Its antioxidant properties stem from the polyhydroxyl structure and gallic acid moiety, which facilitate free radical scavenging, while the presence of phenolic moieties can lead to quinone generation via oxidative sensitivity (56). EGCG exerts antioxidant effects through diverse mechanisms, including hydrogen atom transfer (HAT), electron transfer, and catalytic metal chelation (Figure 2) (57). ROS are metabolically generated by organelles such as mitochondria, peroxisomes, and the endoplasmic reticulum (58). Normally, the antioxidant system efficiently eliminates ROS. However, oxidative stress prompts a shift in signaling pathways, fostering inflammation via pathways like NF-κB, PKC, MAPK, Nrf-2, and PI3K/Akt (59). EGCG mitigates oxidative stress by modulating these pathways (38, 60).

Moreover, studies have indicated that EGCG exerts a direct antioxidant effect by chelating free transition metals such as iron and copper (61). EGCG functions as a free radical scavenger, acting through two mechanisms: HAT and single electron transfer reaction (SET), in relation to its one-electron reduction potential (62). Additionally, EGCG enhances the activity of phase II enzymes and detoxification enzymes, including catalase, glutathione peroxidase (GPX), superoxide dismutase (SOD), and glutathione S-transferase (63). The regulation of these enzymes is primarily governed by Nrf2, which binds to cis-acting regulatory elements to initiate the gene expression of antioxidant enzymes (64). Furthermore, EGCG attenuates excessive levels of NO generated by inducible nitric oxide synthase (iNOS) (65). NO plays a crucial role in various physiological processes at appropriate concentrations. However, under oxidative stress, NO can act as a pro-inflammatory mediator, generating reactive nitrogen species (RNS) such as peroxynitrite (66). Studies have demonstrated that EGCG inhibits iNOS activity, thereby enhancing the bioavailability of NO levels (67). Additionally, EGCG effectively suppresses the activity of xanthine oxidase, an enzyme involved in purine catabolism and uric acid formation, thereby mitigating the associated increase in ROS (68). Moreover, EGCG inhibits the expression of cyclooxygenase-2 (COX-2), an enzyme crucial for fatty acid metabolism that is upregulated during inflammation, particularly in activated macrophages (69).

## 3 Neuroprotective role of EGCG in the context of neurodegenerative diseases

Neurodegenerative disease is a common and growing cause of mortality and morbidity worldwide (70), with 152 million people expected to receive the effects of the disease by 2060 (71), including AD, PD, HD, ALS, and prion diseases (72). Among various forms of dementia, AD exhibits the highest prevalence, accounting for 62%, followed by PD (73). The pathology of AD is characterized by the accumulation of extracellular amyloid β (Aβ) plaques and the formation of intracellular neurofibrillary tangles composed of hyperphosphorylated tau protein (38). Clinical manifestations encompass memory loss, cognitive impairment, personality changes, and in severe cases, hallucinations and seizures (74). PD onset is marked by progressive degeneration of dopaminergic neurons within the substantia nigra, leading to diminished levels of striatal dopamine and its metabolites in the adult brain (75). Clinical features include motor dysfunction, bradykinesia, tremors, gait and balance disturbances, cognitive decline, and disorientation (76). ALS, commonly known as Lou Gehrig’s disease, represents an adult-onset progressive neurodegenerative disorder characterized by selective motor neuron degeneration (77). This degeneration progressively affects both upper and lower motor neurons within the brain and spinal cord. The etiology of ALS remains largely elusive in the majority of cases, with fewer than 10% attributed to specific genetic mutations involving genes such as SOD1, C9orf72, TDP43, and FUS (78). HD arises from an unstable polyglutamine repeat expansion within the first exon of the IT-15 gene, which encodes the 350 kDa huntingtin protein (79). The aggregation propensity of huntingtin fibers contributes to the progressive degeneration of cortical and striatal neurons, alongside the formation of neuronal inclusions containing aggregated huntingtin. Clinical manifestations encompass movement disorders and psychiatric symptoms including chorea, coordination deficits, depression, psychosis, and obsessive-compulsive disorder (80).

While the pathological and clinical presentations of neurodegenerative diseases vary, they share common features including specific pathological alterations within distinct brain regions and the degeneration of various neuronal subtypes. Key factors contributing to neurodegenerative processes encompass the dysregulation of pro-apoptotic proteins, oxidative stress damage, immune-mediated inflammation, mitochondrial dysfunction, and reduced expression of trophic factors (81–83). Here we focus on the crosstalk between EGCG and neurodegenerative diseases in terms of EGCG anti-neuroinflammation and oxidative stress. Neuroinflammation and oxidative stress are intertwined, as inflammation amplifies ROS production while ROS, in turn, exacerbate inflammation (84). ROS can directly activate the NF-kB transcription factor pathway, promoting the synthesis of inflammatory cytokines (85). Given the multifactorial nature of neurodegenerative pathologies, the emergence of novel therapeutic strategies is imperative. The antioxidant properties and neuroprotective effects of EGCG have garnered significant attention from researchers worldwide, positioning it as a promising treatment for neurological disorders and a cytoprotective agent. In this section, we delve into the role of EGCG in mitigating oxidative stress and chronic neuroinflammation in two prevalent neurodegenerative diseases: AD and PD.

### 3.1 Alzheimer’s disease

Neurodegenerative disease affects an estimated 24 million individuals globally, with AD being the most prevalent disease (86). In developed Western nations, individuals aged over 85 exhibit an AD prevalence ranging from 24 to 33%, a figure that escalates with advancing age (87). Given the global aging demographic, AD is poised to become a substantial public health concern over the next two decades and has been identified as a research priority (86). The pathogenic mechanisms underlying AD encompass microglia-induced inflammation, elevated intracellular calcium levels, disruption of antioxidant defense systems, cholinergic dysfunction, overactivation of glutamate receptors, and amplification of the inflammatory response (88). Despite the availability of various medications for managing AD, a definitive treatment remains elusive (89), underscoring the pressing need for research into novel therapeutic approaches and adjunctive therapies. Optimal antioxidant levels in the body have been associated with cognitive preservation, and several studies have demonstrated the neuroprotective effects of catechins, highlighting their potential as adjunctive therapy in select neurodegenerative diseases. These effects rely on the anti-inflammatory and antioxidant properties of catechins (90). Moreover, multiple studies have established a correlation between tea consumption, reduced risk of severe cognitive impairment, and a lower prevalence of AD.

#### 3.1.1 Observational epidemiologic study of green tea consumption and risk of AD

Moeko Noguchi-Shinohara et al. conducted a 2-year follow-up survey of 490 subjects over 60 years of age with cognitive performance and blood tests. Even after correcting for potential confounders, drinking green tea was found to significantly reduce the chance of cognitive deterioration (91). In a questionnaire-based study of 1,003 Japanese participants aged 70 or older, Shinichi Kuriyama et al. discovered a correlation between higher green tea drinking and a lower prevalence of cognitive impairment (32). A brief analysis of tea consumption and prevalence of AD in different country regions by Fernando et al. revealed that countries with higher intake of tea, such as Japan, China, and India, had lower prevalence of AD, whereas European and American countries with lower intake of tea had higher prevalence of AD (92). Although epidemiological data favorably show a negative relationship between drinking tea and the preponderance of AD in that part of the country, any correlation between tea consumption and AD prevalence should be evaluated with caution because the effects of racial differences, dietary preferences, and lifestyle cannot be excluded (92). Yang Yuhuan et al. conducted a questionnaire survey to gauge the cognitive function of seniors 60 years of age and older in the Huangshi community in order to better understand the prevalence of mild cognitive impairment (MCI) and its influencing factors (93). The survey data were tested by chi-square test and it was concluded that the prevalence of MCI was lower in occasional tea drinkers, which may be related to the caffeine and catechins contained in tea, caffeine can reduce the level of Aβ in the brain, which is beneficial for improving cognitive function, while catechins have strong antioxidant capacity, but the study did not prove the relationship between tea drinking and AD prevalence. Wang, Ziqi et al. performed the Mini-Mental State Examination (MMSE) for the assessment of cognitive function in 870 people aged 90 years or older, and cardinality testing of the collected data revealed that the mild cognitive index was significantly different from normal in those who regularly consumed animal oils and legumes (94). In contrast, no significant differences were found for the other 10 foods, including tea, in both the unadjusted and adjusted models (94). Numerous studies have demonstrated the potential of tea consumption to mitigate cognitive decline in older adults; however, experimental evidence supporting its efficacy in AD is lacking (95). Controlled studies examining AD cases have not yielded significant findings regarding tea consumption, thus limiting the inference of beneficial effects of green tea catechins solely based on AD pathogenesis and *in vitro* studies (96). Despite this, the observed efficacy of green tea in AD surpasses initial expectations, warranting further investigation into the specific role of catechins in AD patients.

#### 3.1.2 Experimental studies and mechanisms of AD

Given that Aβ aggregation is recognized as a pivotal factor in the pathogenesis of AD and its impact on the human nervous system, Mahsa Amirpour et al. investigated the neuroprotective potential of green tea in a streptozotocin (STZ)-induced AD model. Their study examined the effects of green tea on cognitive decline, inflammation, and oxidative stress (97). The findings demonstrated that the active compounds present in green tea could mitigate cognitive impairment and ameliorate learning and memory deficits associated with STZ injection (81). Furthermore, green tea may reduce the risk of AD through antioxidative and anti-inflammatory pathways, thus positioning it as a potential preventive intervention (90) (Table 1).

**Table 1:** Specific benefits and mechanisms of action of EGCG in AD.

Animal model	EGCG administration	Outcome measures	Neuroprotective mechanisms	Publication
Aβ 25-35-induced AD rat model.	EGCG (100, 250 or 600 mg/kg/d) by gavage for 9 weeks.	Decreased Tau hyperphosphorylation in the hippocampus; inhibited BACE1 expression and activity as well as Aβ1-42 expression; increased Ach by reducing AchE activity.	Antioxidative stress.	(98)
APP/PS1 transgenic mice (AD model).	EGCG (50 mg/kg) by gavage for 4 months.	Reduced cognitive deficits in AD model mice; improved brain dendritic integrity and synaptic protein expression levels; inhibited microglia activation and reduced pro-inflammatory cytokines (IL-1β); reduced β-amyloid (Aβ) plaques in the hippocampus.	Anti-inflammatory; neuroprotective; anti-amyloidogenic.	(99)
SAMR1 and SAMP8 mice.	EGCG (5 or 15 mg/kg/d) by gavage for 60 days.	Alleviates deterioration of cognitive function; reduced brain NEP levels and decreased accumulation of Aβ.	N/A	(100)
APP/PS1 mice.	EGCG (40 mg/kg/d) orally for 3 months.	Reduces synaptic deficits; reduces neuroinflammation and Aβ plaque accumulation; enhances learning ability and spatial memory.	N/A	(101)
APP/PS1 mice.	EGCG-containing (10 mg/mL) drinking water for 5.5 months.	Restoration of mitochondrial respiration rate, MMP, ROS production, and ATP levels; reduction in toxic levels of brain Aβ.	Antioxidant; reduces mitochondrial dysfunction.	(102)
APP/PS1 mice	EGCG (30 mg/kg/d) by gavage for 90 days.	Reduced brain parenchyma and cerebrovascular Aβ deposition; increased expression of nonamyloidogenic soluble APP-α and α-secretase candidate proteins, as well as decreased expression of amyloidogenic soluble APP-β and β-secretase proteins; alleviated synaptic toxicity, neuroinflammation and oxidative stress.	Anti-neuroinflammatory; antioxidant stress.	(103)
Aβ injection induces AD rat model.	Intraperitoneal injections of EGCG (10 mg / kg) were administered for 3 weeks (every other day).	Reduces Aβ accumulation; restores motor coordination and memory.	N/A	(104)
LPS-induced neuroinflammation and memory impairment in mice.	EGCG (1.5 mg/kg or 3 mg/kg) was administered orally for 3 weeks.	Prevented memory damage and neuronal apoptosis; inhibited elevated Aβ levels and APP and β-site APP cleavage enzyme 1 expression; prevented astrocyte activation; decreased levels of cytokines (TNF-α, IL-1β, GM-CSF, ICAM-1, and IL-16); reduced iNOS and COX-2 expression.	Anti-neuroinflammatory; antioxidant stress.	(105)
SAMP8 mice	EGCG (5 or 15 mg/kg/d) orally for 8 weeks.	Improves spatial learning ability and memory impairment; reduces levels of Aβ1-42 and BACE-1; prevents hyperphosphorylation of tau.	N/A	(106)
APP/PS1 mice	EGCG (2 mg/kg/d) orally for 4 weeks.	Improved cognitive impairment; reduced Aβ and APP expression and inhibited neuronal apoptosis; activation of TrkA signaling and inhibition of p75NTR signaling.	Adjust the TrkA/p75NTR signal balance.	(107)
APP/PS1 mice	EGCG (2 or 6 mg/kg/d) orally for 4 weeks.	Improves learning and memory deficits; decreases hippocampal levels of IRS-1pS636 and Aβ42; inhibits TNF-α/JNK signaling; increases Akt and glycogen synthase kinase-3β phosphorylation in the hippocampus.	Attenuates central insulin resistance.	(108)
Tg APPsw transgenic mice	Intraperitoneal injection of EGCG (20 mg / kg/d) for 60 days.	Promotes APP for nonamyloidogenic processing; reduces cerebral amyloidosis.	N/A	(109)
STZ-induced AD mouse model.	EGCG (10 mg/kg/d) by gavage for 4 weeks.	Reduces cognitive impairment; reverses AChE activity, GPX activity, NO metabolites, and ROS levels.	Antioxidant stress.	(110)

**Figure 3:**
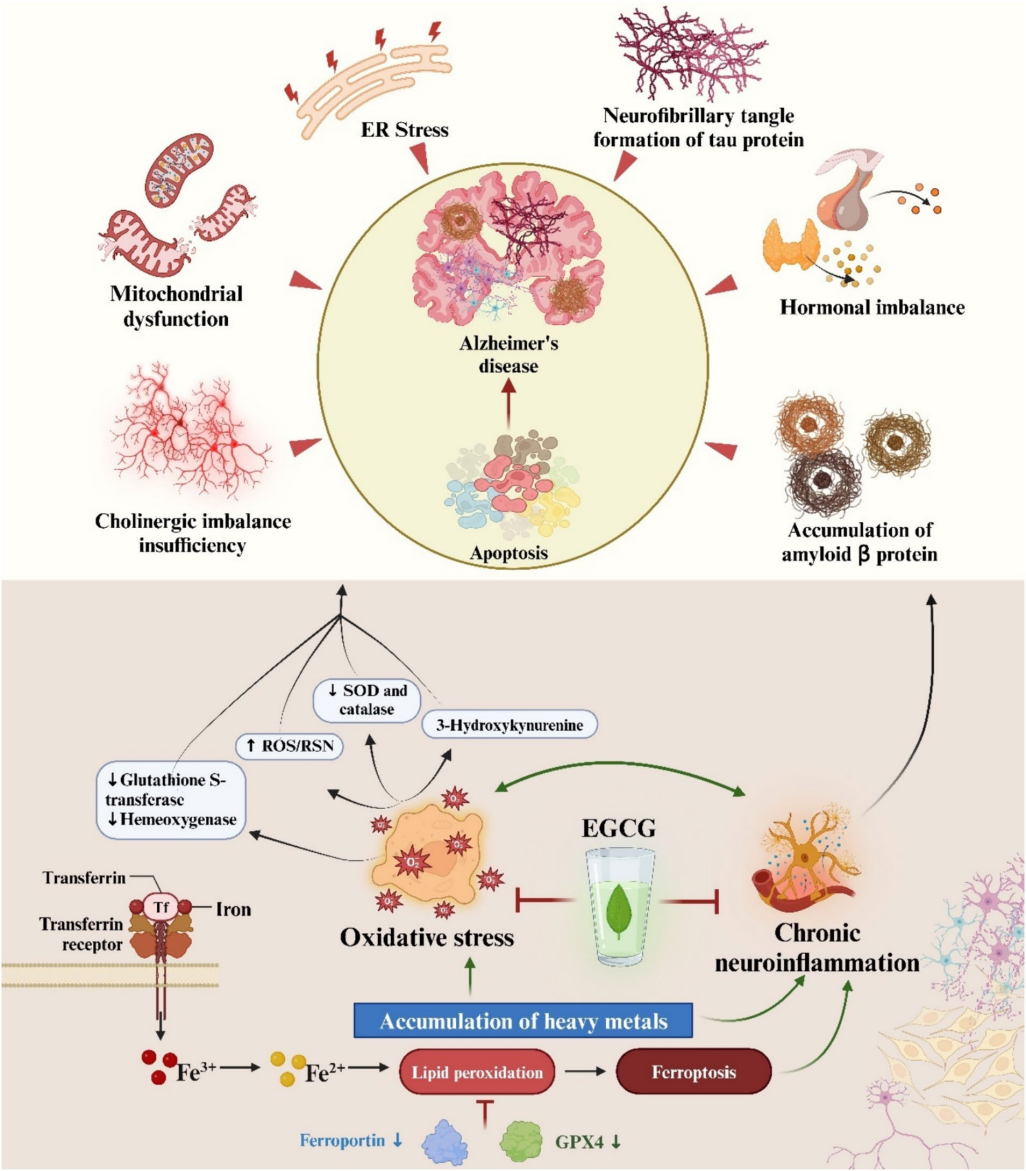
The multifactorial pathophysiology of Alzheimer’s disease is depicted in an illustration. Furthermore, epigallocatechin-3-gallate is highlighted as a potential therapeutic intervention for AD, attributed to its ability to counteract oxidative stress and chronic neuroinflammation.

Tingting Chen et al. used mice as a model to demonstrate that the polyphenolic compounds EGC and ECG effectively alleviated Aβ40 aggregation and protofibrillar toxicity by chelating Cu^2+^ and Zn^2+^ and reduced ROS production, thereby mitigating Cu^2+^-Aβ40 and Zn^2+^-Aβ40induced neuronal toxicity (111). The results showed that tea polyphenols had significant beneficial effects on different aspects of AD pathology (112). Among them, catechin ECG had the most significant effect due to the therapeutic effect of ECG through the BBB, reducing Aβ plaques in the brains of APP/PS1 mice and thus protecting neurons from damage (111). Therefore, the potential of catechins to prevent or improve AD symptoms was laterally demonstrated (111). Lee JW et al. found that EGCG reduced Aβ1-42-induced memory dysfunction by altering the secretion of α-secretase, in addition to EGCG inhibiting Aβ1-42-induced apoptosis (113). These findings imply that EGCG may be a useful tool for delaying the start or progression of AD (Figure 3).

#### 3.1.3 EGCG anti-neuroinflammatory activity in AD

Neuroinflammation as a pathogenesis of AD has been confirmed by numerous studies. It has been found that cerebrospinal fluid levels of pro-inflammatory factors such as IL-1β, IL-6, and TNF-α are high in AD patients and increase with disease progression (114, 115). In addition, microglia, which play an important role in chronic neuroinflammation, are also involved in this process. Microglia resist the onset and progression of AD by degrading Aβ and tau. However, Aβ in turn activates microglia through TLRs to release pro-neuroinflammatory mediators. In the early stages of AD development, neuroprotective phenotypic microglia appear around Aβ plaques (116, 117). However, in late AD pathogenesis, elevated expression of proinflammatory factors will result in the emergence of microglia with a proinflammatory phenotype and a decrease in their phagocytic activity (118, 119). Pro-inflammatory microglia drive tau proliferation and toxicity by promoting neuroinflammation, such as activation of NLRP3 inflammasomes or induction of NF-kB signaling (23). Defective microglial autophagy leads to dysregulation of lipid metabolism, which increases the pathology of tau within neurons further exacerbating AD (23).

Numerous studies have shown that EGCG treatment of AD is associated with chronic neuroinflammation induced by microglia of anti-inflammatory phenotype (105). Wei et al. conducted *in vitro* experiments demonstrating that EGCG effectively suppressed the expression of TNFα, IL-1β, IL-6, and iNOS while concurrently restoring intracellular antioxidant levels, including Nrf2 and HO-1. These actions counteracted the pro-inflammatory effects of microglia (120). Furthermore, EGCG inhibited the secretion of pro-inflammatory factors from Aβ-induced pro-inflammatory microglia phenotypes and attenuated microglial neurotoxicity (121). Importantly, EGCG also mitigated Aβ-induced cytotoxicity by attenuating ROS-mediated NF-κB activation and MAPK signaling pathways, including JNK and p38 signaling (121). *In vitro* investigations have demonstrated that Aβ deposition significantly diminishes following intraperitoneal injection of EGCG at a dose of 20 mg/kg or oral administration of EGCG at 50 mg/kg in drinking water (109, 122). Similarly, Li et al. observed a substantial reduction in Aβ deposition in the frontal cortex (60%) and hippocampus (52%) following oral administration of EGCG at a dose of 20 mg/kg/day for 3 months in an AD mouse model (123). Furthermore, recent findings by Lee et al. revealed that EGCG attenuated LPS-induced memory impairment and neuronal apoptosis, concomitant with a reduction in the expression of inflammatory cytokines TNF-α, IL-1β, and IL-6 (105). These results align with *in vitro* observations, suggesting that EGCG holds promise as a therapeutic agent for neuroinflammation-associated AD.

#### 3.1.4 EGCG antioxidant activity in AD

The brain is particularly vulnerable to oxidative damage due to its high content of easily oxidizable lipids, elevated oxygen consumption rates, and limited antioxidant defense mechanisms. Age-related increases in brain oxidation contribute to the recognized risk of AD (124). Under normal physiological conditions, SOD catalyzes the conversion of superoxide anions to hydrogen peroxide, thereby safeguarding cells against free radical assault. However, in the presence of elevated levels of certain metal ions such as Fe and Cu, SOD can convert hydrogen peroxide to the more hazardous hydroxyl radical (125). Notably, AD patients exhibit heightened SOD activity, diminished glutamine synthetase activity, and elevated lipid peroxidation, collectively resulting in heightened oxidative stress and accumulation of free radicals. Free radicals inflict damage upon biofilms, disrupting the intracellular milieu and precipitating cellular senescence and demise (126). Peroxidation of impaired lipids results in ribonucleic acid inactivation, prompting DNA and RNA cross-linking and instigating DNA mutations (127). Decomposition of peroxidized lipids yields aldehydes, such as acrolein, which react with phosphoric acid and proteins to generate lipofuscin (128). Accumulation of lipofuscin in the brain contributes to cognitive impairment (129). Furthermore, mitochondrial dysfunction and oxidative stress in AD patients are intricately intertwined, with evidence indicating mutual exacerbation, culminating in AD pathogenesis (130).

Numerous studies have delineated the involvement of increased oxidative stress in AD pathogenesis, and highlighted the potential of EGCG’s antioxidant properties in mitigating this process (131, 132). Abdul M. Haque et al. observed that long-term administration of green tea catechins to AD model mice significantly ameliorated cognitive impairment, accompanied by reduced ROS levels and enhanced antioxidant capacity in the hippocampus and cortex (133). Similarly, Regina Biasibetti et al. investigated the effects of oral EGCG administration (10 mg/kg/day) for 1 month in a rat model of dementia, revealing cognitive deficits reversal and notable reductions in ROS levels and NO production (110). Catechins exert their antioxidative effects by scavenging free radicals and chelating metal ions such as Fe and Cu, thereby reducing ROS production. This dual action mitigates oxidative stress in both peripheral and brain tissues, thereby inhibiting further deterioration of cognitive deficits-associated behaviors (134). Mitochondrial dysfunction enhances ROS generation via the NADPH oxidase pathway (135). EGCG reinstates mitochondrial respiration rate, ATP levels, ROS levels, and membrane potential (102). Its antioxidant properties scavenge ROS production and safeguard against mitochondrial damage (136). Furthermore, EGCG treatment mitigates neuronal apoptosis triggered by endoplasmic reticulum stress subsequent to Aβ exposure. The inflammatory response to neuronal injury induced by various stimuli culminates in the release of pro-inflammatory cytokines and cytotoxins, further exacerbating oxidative stress (137). Numerous studies have demonstrated EGCG’s protective effects against lipopolysaccharide-induced memory impairment and inflammatory responses (105, 138). Through mechanisms associated with protein kinase C (PKC), which facilitates the generation of nontoxic soluble peptide APPβ (sAPPβ) and cell survival, catechins may exert an influence on AD (139, 140). Levites et al. reported that EGCG (1–5 μM) enhances sAPPβ production from PC12 and human neuroblastoma cells (141).

### 3.2 Parkinson’s disease

PD follows AD as the second most prevalent neurodegenerative disorder affecting middle-aged and elderly individuals. While PD is uncommon before the age of 50, its incidence escalates markedly with advancing age, peaking between 70 and 85 years, afflicting 7 to 10 million individuals worldwide (142, 143). Pathologically, PD is characterized by the degeneration and loss of dopaminergic neurons within the substantia nigra pars compacta, accompanied by the formation of eosinophilic inclusion bodies known as Lewy bodies within the residual neurons. These alterations disrupt the balance between dopamine and cholinergic neurotransmitters, culminating in aberrant motor function within the basal ganglia. The resultant motor and non-motor symptoms include postural reflex deficits, bradykinesia, muscular rigidity, gait disturbances, and resting tremor (144, 145). The episodic nature of PD in most cases suggests a multifactorial etiology involving genetic susceptibility and environmental influences. While the precise pathogenesis remains elusive, current hypotheses implicate abnormal aggregation of α-synuclein, mitochondrial dysfunction, calcium dyshomeostasis, oxidative stress, and neuroinflammation (146).

#### 3.2.1 Observational epidemiologic study of green tea consumption and risk of PD

In order to determine the relationship between PD incidence and tea consumption, Quintana et al. examined a total of 12 studies from 1981 to 2003, comprising 2,215 cases and 145,578 controls. Their analysis revealed that tea consumption can prevent PD and that this protective effect is more pronounced in the Chinese population (147). In order to study the non-hereditary factors associated with PD, Hosseini Tabatabaei N. et al. used a sample of 150 people, including 75 PD patients and 75 people as controls, and showed that tea intake was protective against PD and that adherence to daily tea consumption reduced the risk of PD by 80% (148). A case–control study was conducted by Harvey Checkoway et al. By studying and counting PD cases (*n* = 210) and controls (*n* = 347), it was found that people who drank two or more cups of green tea per day had a reduced incidence of PD compared to those who did not drink green tea (149). According to research by E-K Tan and colleagues, drinking one unit of tea (3 cups per day for 10 years) would result in a 28% decrease in the incidence of PD (150). The effects of tea consumption on 60 patients with idiopathic PD were examined by Chahra CD et al. According to the study’s findings, PD patients who drank tea in addition to traditional medication experienced improvements in their non-motor symptoms and depression (143). Boris Kandinov et al. also demonstrated that drinking tea and smoking delayed the age of PD attacks, while drinking coffee may have the opposite effect (151). Observational epidemiological studies in PD have more experimental data demonstrating a protective effect of green tea compared to AD, and even though epidemiological findings support the beneficial effects of tea consumption, some have not yet provided clear evidence. Therefore, more research is required to determine the connection between drinking tea and the risk of PD.

#### 3.2.2 Experimental studies and mechanisms of PD

Pathological accumulation of metal ions or a rapid increase in monoamine oxidase B (MAO-B) activity can induce endogenous dopamine (DA) oxidation, leading to α-synuclein aggregation, mitochondrial dysfunction, and other factors contributing to the heightened incidence of PD. Consequently, mitigation strategies involve the use of ROS scavengers, DA oxidation inhibitors, MAO-B inhibitors, and DA quenchers (152). Zhou et al. demonstrated that catechins can impede DA oxidation by inhibiting enzymes and metal ions. Furthermore, they inhibit MAO-B activity, detoxify ROS, DA quenchers, and harmful DA oxidation byproducts, while regulating the Nrf2-Keap1 and PGC-1 pathways. These findings underscore the inhibitory effects of tea polyphenols on DA-related toxicity (153). In a study by Shyh-Mirnin Ph.D. et al., the influence of EGCG on MAO-B enzyme activity in the adult rat brain was investigated, revealing a decrease in MAO-B enzyme activity (154).

PD primarily affects dopaminergic neurons in the substantia nigra pars compacta (SNpc) region of the brain (155). The neurotoxins 1-methyl-4-phenyl-1,2,3,6-tetrahydropyridine (MPTP) or 6-hydroxydopamine (6-OHDA) specifically damage this brain region, resulting in the loss of dopaminergic neurons (156). This neuronal loss leads to disrupted neural firing patterns and impaired motor control (157). Weinreb et al. investigated the impact of pretreatment with tea extract (0.51 mg/kg) and the tea polyphenol EGCG (2.10 mg/kg) on dopamine neurogenesis loss in the substantia nigra of MPTP-induced PD mouse models (158). Their study revealed a considerable mitigation of neurogenesis loss (158). Siddique Y. H. et al. examined the effects of EGCG in an α-synuclein (h-αS) transgenic Drosophila model of PD, analyzing statistical data and markers of changes in climbing capacity, lipid peroxidation, and apoptosis (159). Their findings demonstrated that various concentrations of EGCG (0.25, 0.50, and 1.0 g/mL) substantially delayed the loss of climbing ability in Drosophila, while reducing oxidative stress and apoptosis (159).

In a study by Tingting Zhou et al., a PD mice model induced by MPTP was utilized to investigate the potential therapeutic effects of EGCG for PD. The results demonstrated that EGCG administration ameliorated impaired locomotion behavior in MPTP-treated mice and protected tyrosine hydroxylase-positive cells in the substantia nigra pars compacta from MPTP-induced toxicity (160). Additionally, following EGCG treatment, flow cytometric analysis revealed an increase in the CD3 + CD4+ to CD3 + CD8+ T cell ratio in peripheral blood of MPTP-treated mice. Furthermore, EGCG appeared to downregulate the expression of inflammatory mediators such as TNF and IL-6 in serum (160). These findings suggest that EGCG may confer neuroprotective effects in MPTP-induced PD mice models, potentially by modulating peripheral immune responses.

Current understanding of PD pathogenesis implicates neurofilaments, synaptic vesicle proteins, and ubiquitinated α-synuclein as primary contributors to the disease pathology (161). Additionally, Lewy bodies may exacerbate the release of free radicals, excessive nitric oxide synthesis, microglia-mediated inflammation, and disruption of protein degradation pathways, further exacerbating the pathophysiology (162). Specific beneficial effects and mechanisms of action of EGCG in PD are summarized in Table 2. In conclusion, EGCG exhibit diverse pharmacological activities in PD by modulating gene expression and interfering with signaling pathways (172). Despite substantial experimental evidence supporting this notion, challenges such as low solubility, limited bioavailability, and BBB impermeability hinder efficient delivery of EGCG to the brain and impede clinical translation (173). Overcoming these obstacles necessitates cross-sectional studies aimed at elucidating chemical modification strategies and optimizing drug delivery mechanisms to enhance their therapeutic efficacy.

**Table 2:** Specific benefits and mechanisms of action of EGCG in PD.

Animal model	EGCG administration	Outcome measures	Neuroprotective mechanisms	Publication
MPTP-induced PD mouse model.	EGCG (50 mg/kg/day) gavage administration for 20 days.	PD mice recovered motor behavior; increased the CD3CD4 to CD3CD8 T-lymphocyte ratio in the peripheral blood; and decreased the inflammatory factor (TNF-α and IL-6) expression in the serum.	Anti-neuroinflammatory.	(160)
LPS (substantia nigra injection)-induced PD rat model.	EGCG-Loaded Liposomes 2 μL/d (12.5 μM) was administered for 14 days.	Recovery of dyskinesia in PD rats; reduction of TNF-α production in the brain substantia nigra region; prevention of BV-2 activation.	Anti-neuroinflammatory.	(163)
Paraquat-induced TH > dj-1-β-RNAi/+ *Drosophila melanogaster* flies (PD Drosophila model)	Feed 0.5 mM EGCG for 15 days.	Drosophila restored lifespan and locomotor activity, with decreased lipid peroxidation and neurodegeneration.	Antioxidative stress.	(164)
Rotidone (ROT)-induced PD rat model.	Intravenous EGCG (100 or 300 mg/kg/d) for 21 days.	NO levels and lipid peroxidation were reduced; SDH, ATPase, and ETCase activities, and catecholamine levels were elevated; and levels of neuroinflammatory and apoptotic markers were reduced.	Antioxidant effects; prevention of mitochondrial dysfunction; anti-neuroinflammatory effects; anti-apoptotic effects.	(165)
MPTP-induced PD mouse model.	EGCG (2 and 10 mg/kg/day) gavage administration for 10 days.	Prevention of nigrostriatal dopamine neuron death; restoration of striatal dopamine and tyrosine hydroxylase protein levels; elevation of striatal antioxidant enzymes SOD and catalase activity.	Antioxidant; iron chelate.	(158)
MPTP-induced PD mouse model.	EGCG (2 and 10 mg/kg/day) gavage administration for 10 days.	Reduced neurotoxicity in PD mice; restored rotational latency; increased striatal dopamine concentration and nigral ferritin expression.	Antioxidative stress.	(166)
α-Synuclein preformed fibers (α-syn-PFFs)-induced PD mouse model.	Intraperitoneal injection of EGCG (10 mg/kg/day) for 7 days.	Reduces anxiety-like behavior and dyskinesia in mice; reduces neuronal degeneration and accumulation of p-α-syn in Lewy bodies and Lewy neurons; reduces expression of pro-inflammatory cytokines (IL-6, IL-1, and TNF-α) while promoting expression of anti-inflammatory cytokines (TGF-β, IL-10, and IL-4).	Anti-neuroinflammatory.	(167)
MPTP-induced PD mouse model.	EGCG (25 mg/kg) was administered by gavage for 1, 2, 4 and 7 days.	Prevents loss of TH-positive cells in the SN and loss of TH activity in the striatum; maintains HVA levels in the striatum; decreases nNOS expression in neurons.	Antioxidative stress.	(168)
MPTP-induced PD mouse model.	Intraperitoneal injection of EGCG (10 mg/kg or 50 mg/kg per day) for 14 days.	Reduced neuronal death rate and iNOS expression.	Antioxidative stress.	(169)
MPTP-induced PD mouse model.	EGCG (25 mg/kg/day) gavage administration for 7 days	Increased rotational latency; elevated striatal dopamine concentration; and higher substantia nigra ferritin expression.	Reduction of oxidative stress; iron-export protein ferroportin in substantia nigra.	(166)
LPS -induced PD rat model.	Intraperitoneal injection of EGCG (10 mg/kg/d) for 7 days.	Decreased expression of TNF-α and NO; increased levels of dopamine neurons.	Anti-neuroinflammatory; anti-oxidative stress.	(170)
MPTP-induced PD mouse model.	EGCG (25 mg/kg/day) gavage administration for 6 days	Protected tyrosine hydroxylase (TH)-positive cells in the substantia nigra (SN) and TH activity in the striatum; reduced nNOS expression in the substantia nigra and neuronal nNOS expression.	Antioxidative stress.	(168)
L-DOPA and carbidopa-induced PD rat model.	Only one oral dose of EGCG (25 mg/kg).	Restores striatal dopamine accumulation; reduces glutamate-induced oxidative cytotoxicity by inactivating the NF-kB signaling pathway; reduces neuronal death.	Antioxidative stress; COMT inhibiton.	(171)

#### 3.2.3 EGCG anti-neuroinflammatory activity in PD

Numerous studies have established neuroinflammation as a significant etiological factor in PD, playing a pivotal role in its early pathogenesis. Notably, activated microglia make substantial contributions to this process. Evidence supporting the involvement of activated microglia-mediated chronic neuroinflammation in PD includes: (1) Pro-inflammatory effects are often observed in activated microglia surrounding dopaminergic neurons, with the degree of microglial activation correlating with dopaminergic endings loss in PD (174, 175). (2) Injured neurons release excessive α-synuclein, activating proinflammatory factors like TNF-α, NO, and IL-1β produced by microglia, thereby modulating chronic neuroinflammation in PD (176, 177). (3) Jmjd3, critical for microglial cell phenotype expression, when inhibited, leads to overactivation of pro-inflammatory microglial responses, exacerbating neuroinflammation and neuronal cell death (178). Additionally, it has been proposed that α-synuclein aggregates exert toxicity on neurons only in the presence of microglia (179, 180). In PD patients, misfolded α-synuclein is released from injured neurons into the extracellular fluid, where it binds to Toll-like receptors (TLRs), Fcγ receptors (FcγR), or nucleotide-binding oligomerization domain-like receptors (NLRPs), further activating microglia (181–184). The proinflammatory cytokines released by activated microglia subsequently activate protein kinase R (PKR), leading to phosphorylation of α-synuclein at Ser129, a process considered of significant pathological importance, particularly in Lewy bodies of PD patients. Moreover, microglia are involved in the clearance of protein deposits, including α-synuclein and Aβ, from astrocytes (185–187). Activation of microglia upregulates MHC I expression on neurons, promoting neuronal presentation of α-synuclein antigen. Subsequently, these neurons are targeted and eliminated by α-synuclein-reactive T cells (187). The emergence of α-synuclein pathology follows microglial activation, suggesting α-synuclein’s pivotal role in PD progression, albeit not as an initiator. Similarly, mounting evidence suggests that the immune response contributes to neuronal death as a cause rather than a consequence (22).

**Figure 4:**
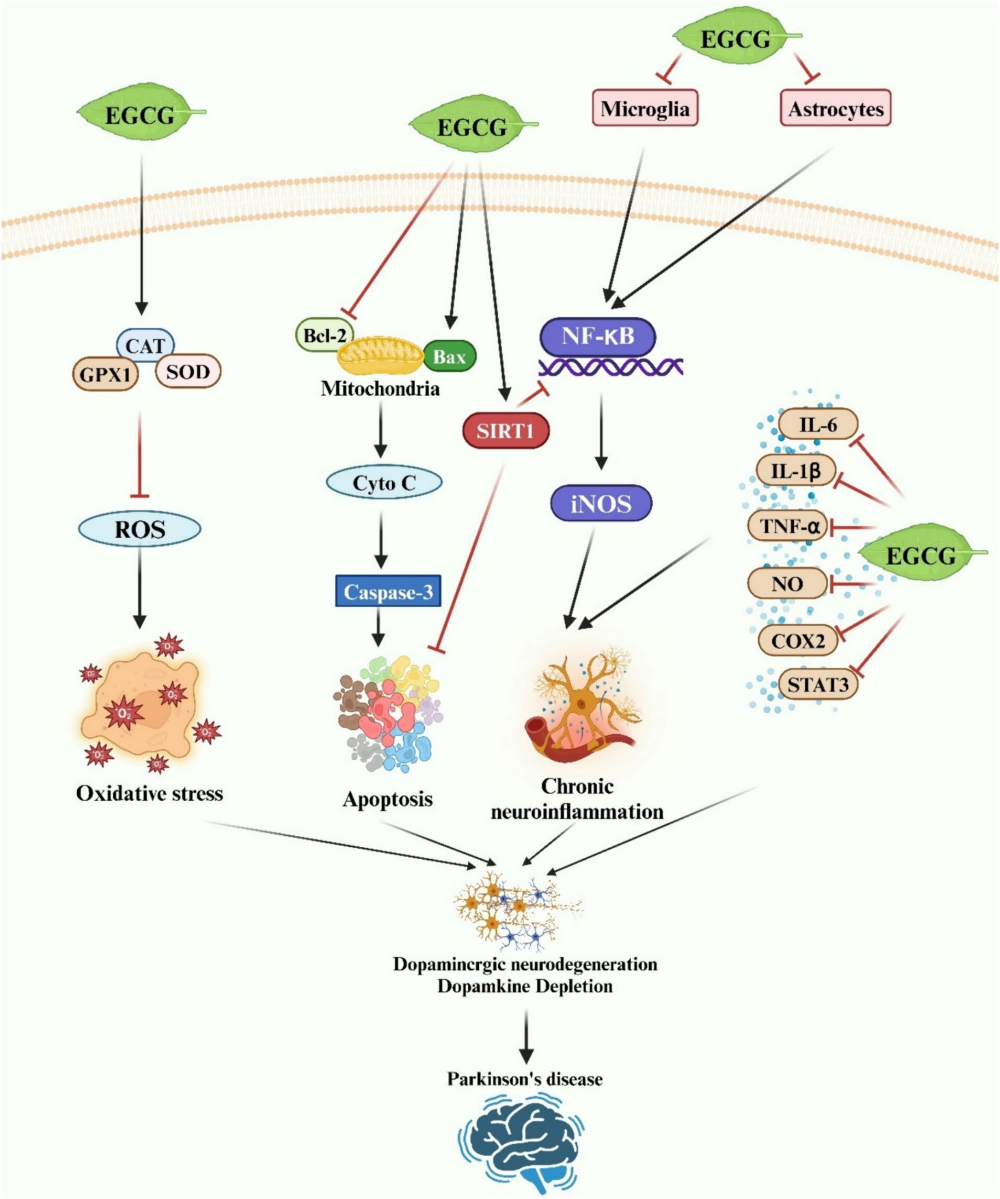
A schematic representation illustrates the neuroprotective effects of EGCG in Parkinson’s disease. EGCG exerts neuroprotection by inhibiting oxidative stress, neuronal apoptosis, and neuroinflammatory responses via diverse molecular mechanisms.

A growing body of evidence suggests that EGCG may impede or postpone the progression of PD by targeting chronic neuroinflammation. EGCG exhibits potent anti-inflammatory activity both *in vitro* and *in vivo*, primarily attributed to its ability to inhibit microglia-induced cytotoxicity (120). *In vitro* studies have demonstrated that EGCG suppresses the secretion of pro-inflammatory factors from LPS-activated microglia by downregulating the expression of iNOS and TNF-α (188). Furthermore, EGCG has been shown to inhibit microglial activation and reduce neuronal damage in SH-SY5Y and rat mesencephalic cultures (188). Gülşen Özduran et al. reported that EGCG restored viability in PD model cells, inhibited apoptosis, and enhanced survival by attenuating 6-OHDA-induced expression of TNF-α and IL-1β in SK-N-AS cells (189). The findings from the *in vivo* study corroborate those observed *in vitro*, further substantiating the potential of EGCG to mitigate the inflammatory response associated with microglia-mediated damage to dopaminergic neurons. Al-Amri et al. demonstrated that EGCG significantly increased the number of TH-immunoreactive neurons in the midbrain of PD model rats by reducing the production of TNF-α and NO (170). Similarly, EGCG liposomes alleviated symptoms in a PD rat model by suppressing the expression of NO and TNF-α in microglia exhibiting an LPS-induced inflammatory phenotype (165). In summary, EGCG shows promise as a therapeutic and prophylactic agent for PD, exerting neuroprotective effects both *in vivo* and *in vitro* through the inhibition of neuroinflammation (Figure 4).

#### 3.2.4 EGCG antioxidant activity in PD

PD patients commonly exhibit reduced mitochondrial complex I activity and increased ROS production (190). This diminished function of proton pumps on mitochondria, coupled with decreased membrane voltage and the opening of permeability channels, initiates the apoptotic process. Deficiency in mitochondrial complex I can result in oxidative stress, heightening neuronal susceptibility to excitotoxic injury. The densely packed substantia nigra is particularly vulnerable to elevated oxidative stress compared to other brain regions. Under normal conditions, H_2_O_2_ generated by dopamine toxicity is neutralized by reduced glutathione, mitigating potential harm. However, in the remaining dopamine neurons of PD patients, ineffective scavenging of H_2_O_2_ may occur due to compensatory mechanisms, including accelerated toxicity production in dopamine metabolism, heightened monoamine oxidase (MAO)-B activity, and reduced glutathione levels (191). Excessive H_2_O_2_ reacts with Fe^2+^ via Fenton chemistry, yielding highly toxic hydroxyl radicals, culminating in lipid peroxidation and apoptosis of nigral neurons. This oxidative stress and mitochondrial dysfunction form a reciprocal relationship, perpetuating a vicious cycle.

The neuroprotective effects of EGCG, attributed to its antioxidant properties, have been observed in PD (Figure 4). Typically, α-synuclein localizes to the mitochondria-associated membrane, and its presence may disrupt mitochondrial function by promoting the formation of the mitochondrial permeability transition pore (mPTP), leading to mitochondrial membrane potential (MMP) loss, subsequent mitochondrial degradation, and ultimately cell death (192). Compounds capable of preserving mitochondrial activity are therefore deemed invaluable in combating PD. EGCG has been shown to safeguard mitochondrial function by preventing Ca2+ influx through voltage-gated calcium channels and mitochondrial Ca2+ uptake via the mitochondrial Ca2+ uniporter (159, 193). Furthermore, *in vivo* studies have demonstrated EGCG’s ability to reduce oxidative stress by decreasing serum protein carbonyls and mitigating neurotoxicity in the MPTP-induced mouse model of PD (166). Similarly, Pinto et al. reported that EGCG improved cognitive dysfunction induced by 6-OHDA in male Wistar rats. 6-OHDA is known to induce ROS generation. EGCG treatment reversed striatal oxidative stress and attenuated immunohistochemical alterations (194). In conclusion, EGCG has been shown to alleviate PD by inhibiting neurotoxin-induced oxidative stress injury both *in vitro* and *in vivo*.

## 4 EGCG bioavailability, toxicity, and safe dose

When evaluating EGCG for clinical therapeutic applications, significant concerns arise regarding its safety, toxicity, and optimal dosage post-treatment. While numerous studies have highlighted EGCG’s beneficial impact on neurodegenerative diseases due to its antioxidant and anti-neurotoxic properties, others have reported adverse effects such as heightened oxidative stress and the generation of toxic EGCG metabolites (195–197). Hence, there is a critical need for systematic investigations into EGCG’s bioavailability, toxicity profile, and appropriate dosing regimens.

### 4.1 Bioavailability of EGCG

EGCG has been extensively investigated for its notable health-promoting effects, with particular focus on its neuroprotective properties. Despite these benefits, the bioavailability of EGCG is limited, posing a challenge for its clinical application in treating neurodegenerative diseases. Following oral administration, EGCG exhibits a mean peak plasma concentration between 1.3 and 2.2 h, a half-life ranging from 1.9 to 4.6 h, and is almost completely metabolized within 24 h (198). Pharmacokinetic studies reveal that merely 0.1% of the ingested EGCG dose reaches detectable levels in the bloodstream at its peak concentration time (Tmax) in healthy individuals (39, 199). This minimal absorption occurs primarily through passive diffusion (paracellular and transcellular diffusion) in the small intestine, while the remaining EGCG reaches the colon to be degraded by intestinal microbial enzymes (198, 200, 201). EGCG undergoes phase II metabolism in enterocytes and hepatocytes following ingestion (199, 202). The polyphenolic hydroxyl structure of EGCG facilitates binding reactions such as methylation, glucuronidation, sulfation, and cysteine binding, contributing to its limited bioavailability (203). Upon entering the colon, EGCG encounters rapid hydrolysis of conjugate groups like glucuronides and sulfates by colonic microbiota. Subsequently, glycosides are released and further catabolized into ring cleavage products and low molecular weight phenolic acids (198, 204). While absorption studies traditionally focus on the small intestine, phenolic acid metabolites degraded by colonic microorganisms constitute approximately 40% of the ingested EGCG, underscoring the significant role of colonic metabolism in EGCG bioavailability.

Keiko Unno et al. demonstrated that EGCG can penetrate the BBB to access the brain parenchyma, influencing neuronal cell proliferation and neurogenesis, thus potentially mitigating neurodegenerative diseases (34). There are two common views on the impact of EGCG bioavailability on neuroprotection. We know that only a small fraction of oral EGCG is absorbed into the circulation. In addition, Shimizu et al. found that oral EGCG accumulates primarily in the gut (50%), with less than 0.01% distributed in the liver, blood, and brain (205). Upon comparison of the distribution of EGCG in mice following oral and intravenous administration, it was observed that the majority of orally administered EGCG entered the bloodstream in its glucuronidated form. Additionally, a significant portion of EGCG accumulated in the small intestine and colon (206). Intravenously injected EGCG was rapidly distributed in an uncoupled state in other tissues such as brain, liver, and lung (207, 208). These findings underscore the notion that while intravenous EGCG achieves rapid tissue penetration, oral administration necessitates absorption through the intestine followed by redistribution to tissues and organs. Thus, intestinal absorption emerges as a critical factor limiting EGCG bioavailability and its potential neuroprotective effects across the BBB.

An alternative perspective posits that the gut harbors a substantial population of immune cells and neural networks, and EGCG has the potential to modulate signaling and functional disruptions in intestinal neuroimmune communication via the brain-gut axis (13, 209–211). This theory underscores the gut-brain axis as pivotal in brain injury, neuroinflammation, and related diseases, with microbiota signaling pathways playing a crucial role in neuroprotection (212, 213). It is known that gut microbes can metabolize EGCG into fission products that are more bioavailable and easier to pass through the BBB to exert neuroprotective effects (214). Moreover, evidence supporting EGCG’s neuroprotective effects via BBB-mediated anti-neuroinflammation and reduction of oxidative stress includes: (i) EGCG enhances dopamine neuron activity in the gut, decreases serotonin levels in the colon, and increases hippocampal 5-hydroxytryptamine levels by enhancing intestinal permeability (215–217). (ii) EGCG alleviates intestinal inflammation and repairs the intestinal barrier by altering the gut microbiome. The alteration of the gut microbiome ultimately results in the alleviation of neuroinflammation and neurodegenerative diseases by affecting physiological processes such as immune cell development and migration, amyloid deposition, BDNF and NMDA signaling (218–220). (iii) EGCG also impacts the metabolome of gut microbes, influencing short-chain fatty acids, secondary bile acids, and tryptophan-related metabolites (221, 222). These metabolites traverse the BBB and modulate the host’s nervous system.

### 4.2 Toxicity of EGCG

Despite its limited oral bioavailability, EGCG can induce toxicity, particularly when administered in fasted states or at high doses. Numerous studies have questioned whether EGCG has a clinical therapeutic role, as well as concerns about EGCG toxicity during treatment of various neurodegenerative diseases. Multiple system atrophy (MSA) is a rare neurodegenerative disorder characterized by neuronal loss and gliosis in various regions of the CNS, including the striatum, olivocerebellum, and central autonomic structures (223). A histopathological hallmark of MSA is the presence of oligodendrocyte cytoplasmic inclusions containing misfolded and aggregated α-synuclein (223, 224). EGCG has been shown to inhibit α-synuclein aggregation and mitigate associated toxicity. Johannes Levin et al. conducted a randomized, double-blind, parallel-group, placebo-controlled clinical trial, which demonstrated that 48 weeks of EGCG treatment did not alter disease progression or provide clinical benefit in MSA (225). Two patients discontinued EGCG therapy due to severe hepatotoxicity during the trial (225). The study concluded that elevated transaminase concentrations at therapeutic doses greater than 1,200 mg would cause hepatotoxicity (225). However, the study affirms that EGCG is generally well tolerated in humans and supports the idea that EGCG therapy acting on the α-synuclein oligomer formation may be an effective target for the treatment of neurodegenerative diseases (225). Additionally, numerous animal studies have highlighted adverse effects of EGCG, particularly affecting the liver and kidneys (226–228). We focus on the hepatotoxicity and nephrotoxicity of EGCG and briefly summarize the other adverse effects of EGCG (gastrointestinal toxicity).

#### 4.2.1 Hepatotoxicity of EGCG

The liver is known to be the major drug metabolizing organ in the human body. Initially, K Nakagawa et al. examined the distribution of EGCG (500 mg/kg body weight) in the body after 1 h of oral administration in rats (227). They observed that EGCG concentrations were highest in the intestine, followed by the liver, with plasma levels approximately one-fourth of those in the liver and notably lower concentrations in the brain (227). Autopsy findings further confirmed EGCG induced hepatotoxicity, correlating the extent of liver damage with dosage, route, and duration of EGCG administration (228). Studies on oral EGCG toxicity have documented varying degrees of hepatotoxicity, ranging from mild elevation in liver enzymes (alanine aminotransferase (ALT) and aspartate aminotransferase (AST)) to severe hepatocellular necrosis and bile duct hyperplasia as therapeutic doses increased (229). Thus, it is evident that the liver is a significant target organ for EGCG toxicity.

Animal studies have shown that the severity of liver injury produced by EGCG treatment is related to dose, administration route, and treatment duration. Balaji Ramachandran et al. investigated the relationship between the adverse effects of EGCG treatment with dose and administration route by giving EGCG (108, 67.8, 21.1, and 6.6 mg/kg/d) orally or intraperitoneally to mice (230). Subcutaneous injection of 108 mg/kg EGCG resulted in severe hepatic parenchymal congestion, hepatocellular balloon-like degeneration, kupffer cell hyperplasia, and calcification (acute hepatitis); serum levels of bilirubin, AST, ALT, and ALP were markedly elevated, leading to mortality by the 8th day of treatment (230). Mice injected with 67.8 mg/kg EGCG subcutaneously exhibited moderate hepatic peritoneal and mild lobular inflammation; elevated serum AST and ALT levels were observed, with mortality occurring by day 16 of the experiment (230). In comparison to subcutaneous injection, oral administration of EGCG resulted in lower hepatotoxicity, with significant liver damage observed only in mice receiving 108 mg/kg EGCG orally. Notably, increasing EGCG doses correlated exclusively with hepatic toxicity, ranging from mild periportal inflammation to severe hepatitis (230). Similarly, Dongxu Wang et al. investigated the dose-dependent hepatotoxic effects of subcutaneously injected EGCG (55, 70, and 125 mg/kg/day) in mice (229). Their findings revealed that all mice injected with 125 mg/kg or 70 mg/kg EGCG succumbed within 2 days, showing severe hepatotoxicity characterized by elevated serum levels of ALT, AST, and 4-HNE, along with increased expression of Nrf2 target genes in the liver. Mice injected with 55 mg/kg EGCG exhibited hepatotoxic effects but survived the duration of the study (229). It has also been demonstrated that subcutaneous injection of 45 mg/kg/day of EGCG represents the maximum tolerated dose in mice, with long-term administration at this dose showing no impact on the body’s oxidative defense mechanisms (231). However, injections of 55 or 75 mg/kg/day of EGCG induced hepatotoxicity in mice, accompanied by inhibition of hepatic antioxidant enzymes and increased nuclear distribution of Nrf2 (231). Furthermore, repeated injections of 75 mg/kg/day of EGCG altered the oxidative defense mechanism, significantly reducing levels of SOD, catalase, and GPX (231). Subcutaneous injection of EGCG in mice at doses exceeding 100 mg/kg/day induces severe hepatotoxicity and dose-dependent mortality, with higher concentrations leading to accelerated death. This treatment also inhibits Nrf2 target gene expression and diminishes antioxidant defense capacity (231). Similarly, gavage administration of EGCG yielded comparable results: mice exhibited hepatic congestion and a slight elevation in ALT levels after receiving 750 mg/kg/day of EGCG for 5 consecutive days (228). Following gavage of 750 mg/kg/day of EGCG for 7 consecutive days, mice exhibited a significant increase in ALT, MDA, MT, and γH2AX levels in the liver, along with hepatocyte degeneration, resulting in a mortality rate of 75% (232). single gavage of 1,500 mg/kg of EGCG led to a 108-fold increase in ALT levels and an 85% mortality rate among mice (232). Metabolites EGCG-2′-cysteine and EGCG-2″-cysteine were detected in urine following high-dose gavage of EGCG (233). Notably, EGCG administered via diet was well tolerated and demonstrated reduced hepatotoxicity compared to gavage administration in animals (233). Studies administering EGCG to Beagles indicated that fasting increased the likelihood of hepatotoxicity compared to animals that were fed prior to treatment (234). These findings underscore the influence of dose, route of administration, treatment duration, and nutritional status on EGCG-induced hepatotoxicity.

Animal experiments have shown that EGCG induced hepatotoxicity correlates with changes in several oxidative stress markers in the body, including MDA, 4-HNE, MT, γH2AX, and Nrf2 (228, 229, 231, 235). MDA and 4-HNE are products of lipid peroxidation and serve as biochemical indicators of oxidative stress (228). MT and γH2AX are molecular markers associated with oxidative stress. All these biomarkers suggest that hepatotoxicity induced by EGCG treatment is largely induced by oxidative stress (201). Nrf2 functions as a crucial transcription factor in antioxidant defense. Under normal physiological conditions, Nrf2 is sequestered by Keap1; however, during oxidative stress, Nrf2 dissociates from Keap1 and translocates to the nucleus where it binds to antioxidant response elements. This activation of the Nrf2-ARE signaling pathway upregulates the expression of various antioxidant genes such as HO-1, GST, and NADP (H): NQO1 (231). The Nrf2-ARE signaling pathway activates and enhances the expression of downstream antioxidant enzymes, serving as a critical cellular defense mechanism against oxidative stress (236). This pathway, particularly in the liver, is pivotal in mitigating EGCG-induced hepatotoxicity (236). Animal studies have shown that subcutaneous injection of EGCG at 45 mg/kg/day in mice does not impair major hepatic antioxidant defenses but modestly increases hepatic expression of Nrf2 target genes (231). Conversely, injection of 75 mg/kg/day of EGCG inhibits major hepatic antioxidant enzymes while significantly elevating Nrf2 expression and its target genes (231). Injection of 100 mg/kg/day of EGCG notably suppresses the hepatic Nrf2 pathway (231). These findings indicate a biphasic response of Nrf2 to different EGCG doses. In summary, EGCG-induced hepatotoxicity involves the inhibition of major antioxidant enzymes, with the Nrf2 salvage pathway playing a crucial role in mitigating toxicity. However, this pathway becomes inhibited at higher concentrations of EGCG.

#### 4.2.2 Other toxicities of EGCG

Nora O. Abdel Rasheed et al. investigated potential nephrotoxic effects of EGCG treatment in diabetic mice, a crucial concern due to the kidney’s vulnerability in diabetes (237). Diabetic mice injected with 100 mg/kg EGCG daily for 4 days exhibited decreased resistance to oxidative stress, as indicated by elevated NADPH oxidase levels and reduced expression of Nrf2, HO-1, and HSP90 (237). Serum levels of CYS-C and NGAL were significantly elevated, and histopathological analysis confirmed EGCG-induced renal injury in diabetic mice (237). Similarly, another study demonstrated nephrotoxicity in colitis mice treated with green tea extract containing 35% EGCG, evidenced by increased serum creatinine levels (a nephropathy biomarker), and elevated expression of antioxidant enzymes (HO-1 and NQO1) and HSP 90 (238). These findings collectively underscore the potential nephrotoxic effects of EGCG treatment, exacerbated by oxidative stress implicated in diabetes and its complications (239). Thus, caution is advised when considering EGCG supplements for diabetic patients, particularly at high doses.

In addition to nephrotoxicity and hepatotoxicity, numerous studies have documented gastrointestinal toxicity associated with EGCG administration, whether by gavage or in diet, in animal models (240–242). The severity of gastrointestinal effects varied with dosage, ranging from mild gastric erosion and vomiting to severe ulceration, hemorrhage, and epithelial necrosis. Notably, gastrointestinal toxicity was more pronounced in animals administered EGCG via gavage or when fasted, whereas administration via diet, water, or capsule resulted in milder effects (234, 243). In conclusion, treatment with EGCG at high doses or for prolonged duration may have adverse effects, and the above data suggest that the boundary between protective and toxic doses of EGCG may be narrow.

### 4.3 Safe dose of EGCG

Another critical issue is establishing safe dosage levels of EGCG to optimize therapeutic efficacy while minimizing adverse effects. Current clinical studies on EGCG dosages vary widely, and extrapolation from animal dosages to humans is virtually impossible (244–255). Safety data from human studies indicate distinct toxicity thresholds for EGCG consumed as a beverage compared to capsules or tablets, necessitating separate consideration of safe intake levels. Studies have shown that ingestion of up to 676 mg of EGCG in capsules or tablets did not result in significant adverse effects in healthy adults or patients with various conditions (256). In addition, liver toxicity has been documented with the intake of 800 mg or 1,200 mg of EGCG (225, 253). However, considering that the pro-health benefits of EGCG are similar to those of nutrients. Jiang Hu et al. used an approach similar to the Institute of Medicine (IOM) nutrient risk assessment to determine the safe intake of EGCG (257). The results indicated that the safe intake of EGCG in capsules or tablets for adults is 338 mg/day (257). This safe dose is consistent with the dose derived from animal data (322 mg/day) and is consistent with recent doses proposed by Yates et al. (258) and Dekant et al. (195). Regarding the toxicity threshold for EGCG intake in the form of beverages, the highest reported intake level of EGCG was 704 mg/day with no apparent adverse effects (245). For the current study, it is still uncertain what the standardized safe intake level of EGCG is, as the data currently available from human clinical studies may vary in terms of design, duration, and subject populations. However, the results of the current analyses suggest that diluting and/or slowing the rate of systemic administration of EGCG often appears to be better tolerated by the body. Even so, careful calculation of daily EGCG intake is important when EGCG is used as a dietary supplement. When other EGCG sources are available, EGCG intake may require health-based guidance. The use of EGCG as a clinical agent for neurodegenerative diseases still requires further evaluation of toxicity and dosage.

## 5 Conclusion

In conclusion, this review highlights the significant potential of EGCG, a prominent catechin abundant in green tea, as a therapeutic agent for neurodegenerative diseases. By targeting chronic neuroinflammation and oxidative stress, EGCG demonstrates promising neuroprotective effects in conditions such as AD and PD. Through its antioxidant properties and anti-inflammatory activities, EGCG shows efficacy in mitigating key pathological mechanisms associated with neurodegeneration. The comprehensive exploration of EGCG’s molecular mechanisms, including its modulation of autoimmune responses, nervous-immune system interactions, and inflammatory pathways, underscores its therapeutic relevance in AD and PD. Observational epidemiological studies and experimental investigations provide compelling evidence for EGCG’s neuroprotective effects, supporting its potential as a therapeutic intervention. Furthermore, EGCG’s ability to scavenge free radicals, chelate iron, and attenuate neuroinflammatory processes highlights its multifaceted mechanisms of action. Overall, EGCG emerges as a promising natural compound with the capacity to combat chronic neuroinflammation and oxidative stress, offering novel avenues for the development of neuroprotective strategies in the treatment of neurodegenerative disorders. Further research into EGCG’s therapeutic potential, including clinical trials and mechanistic studies, is warranted to fully elucidate its efficacy and safety profile in neurodegenerative diseases.

## Author contributions

SL: Writing – original draft, Writing – review & editing. ZW: Writing – review & editing. GL: Supervision, Writing – review & editing. MC: Funding acquisition, Supervision, Writing – review & editing.
